# Deep Oxidative Desulfurization of Planar Compounds Over Functionalized Metal–Organic Framework UiO-66(Zr): An Optimization Study

**DOI:** 10.1021/acsomega.3c09971

**Published:** 2024-05-23

**Authors:** Bijan Barghi, Tanel Mõistlik, Anastassia Raag, Maria Volokhova, Indrek Reile, Liis Seinberg, Valdek Mikli, Allan Niidu

**Affiliations:** † Virumaa College School of Engineering, Tallinn University of Technology, Järveküla 75, Kohtla-Järve 30322, Estonia; ‡ National Institute of Chemical Physics and Biophysics, Akadeemia 23, Tallinn 12618, Estonia; § Department of Materials and Environmental Technology, Tallinn University of Technology, Tallinn 19086, Estonia

## Abstract

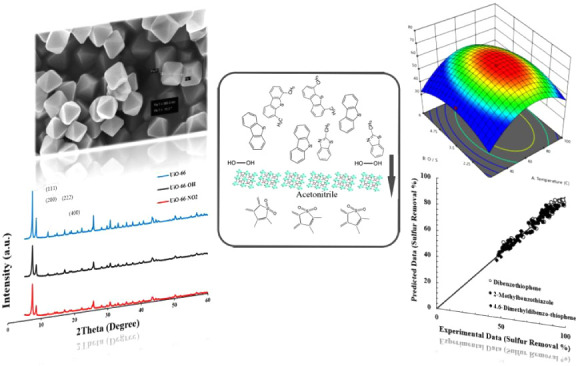

This study aims to determine the catalytic activity and stability of ligand-modified UiO-66 with different functional groups (−NO_2_, −OH) in deep oxidative desulfurization from a model fuel (MF). The planar sulfur compounds included dibenzothiophene (DBT), 2-methylbenzothiazole (2-MB), and 4,6-dimethyldibenzothiophene (4,6-DMDBT) in *n*-dodecane as the fuel phase. The synthesized functionalized metal–organic framework (MOF) samples were characterized by X-ray powder diffraction (XRD), Fourier transform infrared (FTIR), proton nuclear magnetic resonance (^1^H NMR), scanning electron microscopy (SEM), thermogravimetric analysis (TGA), nitrogen adsorption–desorption analysis, and microwave plasma-atomic emission spectrometer (MP-AES). The experiment assessment and desulfurization reaction optimization were carried out by the central composite design methodology. Response surface methodology and analysis of variance were employed to evaluate the individual process factors, their interactions, and sulfur removal responses. The responses showed that the oxidation of the planar compounds declined following the sequence DBT > 2-MB ≫ 4,6-DMDBT for all the MOFs. The findings revealed that at 66.7 °C, 3.0 equiv of oxidative agent over sulfur and 9.7 of MOF over sulfur by weight achieved the highest removal efficiency of 98.68% DBT, 93.23% 2-MB, and 69.32% 4,6-DMDBT for UiO-66-NO_2_ as a catalyst from the model fuel. It was also observed that UiO-66-NO_2_ had a higher efficiency in deep oxidative desulfurization when compared to other UiO-66-based catalysts used in the current study. Under optimal conditions, all the MOFs showed acceptable catalytic activity and reusability after four runs, although gradual loss of activity was observed.

## 1 Introduction

One of the primary sources of air pollution is sulfur oxide emissions into the atmosphere from combustion of fossil fuels that leads to environmental and health problems, so improving deep desulfurization process is essential for producing ultraclean sulfur-free fuel.^1^ Oxidative desulfurization (ODS) is a practical approach for removing planar sulfur compounds,^2^ compared with hydrodesulfurization (HDS) reaction, which may be conducted under mild operating conditions.^3,4^ ODS has a potential to reduce investments in equipment and hydrogen consumption.^5,6^ There are other methods (e.g., photo-oxidation desulfurization (PODS) and oxidative-extractive desulfurization (OEDS)) employed for the deep desulfurization.^7,8^

The ODS process includes oxidation and extraction steps for sulfur compounds. The compounds are oxidized by an oxidant agent; the oxidized sulfurs may then be extracted from the fuel by using an appropriate polar solvent.^9^ The oxidation of sulfur compounds is performed by different oxidant agents such as hydrogen peroxide, oxygen, or *tert*-butyl hydroperoxide.^10−12^ Nevertheless, hydrogen peroxide is the most efficient oxidative agent among the others.^13,14^ For fuel oxidative desulfurization reactions, a variety of catalysts have been applied, including porous materials with titanium,^15^ metal oxides,^16^ boron nitride,^17^ reduced graphene oxide,^6^ activated carbon,^18^ zeolite,^19^ and metal–organic frameworks (MOFs).^20^ MOFs are three-dimensional coordination polymers containing organic linkers and metal nodes, forming an emerging class of porous materials for various applications. These substances exhibit both inorganic and organic flexibility. Additionally, groups that are attached to the linkers and metal node MOFs could be the catalytic active site for reactions.^21,22^ Among MOFs, the porous zirconium terephthalate UiO-66 has been widely used as a catalyst because of its large surface area, tunable porosity, stability, and reusability.^23,24^ Modification of UiO-66(Zr) by adding different ligands, in this case functionalized terephthalic acid, provided a promising route to modifying the chemical and physical characteristics of the MOF;^25−27^ it also could increase the Lewis-basic sites in the structure and make functionalized UiO-66 an appropriate option for adsorption, drug delivery, and heterogeneous catalyst.^28,29^

In previous studies, several metal–organic frameworks have been found to be active catalysts for oxidative desulfurization; however, it was claimed that UiO-66 obtained high sulfur removal efficiency in a shorter period of the process.^30^ Previous reports on adsorption and oxidation efficiency of pristine-, amino-, and nitro-modified UiO-66 are summarized in Table 1:

**Table 1:** Catalytic Oxidation of Planar Sulfur Compounds over MOFs by Using H_2_O_2_

No.	MOF	sulfur compound (removal efficiency % in optimum temperature)	fuel base	temperature (°C)	time (minute)	reference
1	UiO-66	DBT (55%)	*n*-octane	60	120	(23)
2	UiO-66-D^a^	DBT (78%)	*n*-octane	60	120	(23)
3	UiO-66	Th^b^ (47%), BT^c^ (60%), DBT (98%), 4,6-DMDBT (69%)	*n*-octane	30–70	60–180	(24)
4	UiO-66	DBT (93%)	*n*-octane	50	240	(25)
5	UiO-66-MW^d^	DBT (95%)	*n*-octane	50	240	(25)
6	UiO-66	DBT (69%)	*n*-octane	40–70	120	(27)
7	UiO-66-NH_2_	DBT (38%)	*n*-octane	40–70	120	(27)
8	UiO-66-NO_2_	DBT (96%)	*n*-octane	40–70	120	(27)
9	UiO-66-Br	DBT (78%)	*n*-octane	40–70	120	(27)
10	UiO-66	DBT (77%)	*n*-octane	60	120	(28)
11	UiO-66-NH_2_	DBT (68%	*n*-octane	60	120	(28)
12	UiO-66-NO_2_	DBT (90%)	*n*-octane	60	120	(28)
13	UiO-66-g^e^	DBT (96%), 4,6-DMDBT(95%)	*n*-octane	60	120	(28)
14	UiO-66-NH_2_-g^e^	DBT (70%), 4,6-DMDBT(48%)	*n*-octane	60	120	(28)
15	UiO-66-NO_2_-g^e^	DBT (97%), 4,6-DMDBT(96%)	*n*-octane	60	120	(28)
16	UiO-66-NH_2_	DBT (87.5%)	*n*-dodecane	20–100	150	(30)
17	UiO-66	DBT (100%), 4-MDBT (100%), 4,6-DMDBT (100%)	*n*-octane	50	60	(31)
18	UiO-66	DBT (84%), 4,6-DMDBT(50%)	*n*-octane	60	120	(32)
19	UiO-66-free^f^	DBT (100%), 4,6-DMDBT(100%)	*n*-octane	60	120	(32)
20	UiO-66	BT (82%)	*n*-heptane	40	20	(33)
21	UiO-66	BT (64%), DBT (82%), 4-MDBT (63%), 4,6-DMDBT (42%)	*n*-octane	50	240	(34)
22	UiO-66	DBT (100%)	*n*-octane	30	60	(35)

a With defect

b Thiophene

c Benzothiophene

d Microwave advanced synthesis

e Green

f Solvent-free

Thus, far, there has been one report in our previous work, employing UiO-66-NH_2_ as a catalyst on reaction variable assessment in the ODS reaction;^30^ this research employed a more electron-rich ligand, aminoterephthalic acid, to modulate the catalytic activity of nodes and increase the potential for hydrogen bonding in pores via the NH_2_ group.

Currently, a range of diverse catalysts have been identified for ODS reactions, with limited studies involving functionalized MOF UiO-66. There have been no investigations on the process condition optimization of different functionalized UiO-66 (-NO_2_, −OH) as in the ODS process. In this study, pristine UiO-66(Zr), UiO-66-NO_2_, and UiO-66-OH were prepared by a solvothermal procedure for the oxidative desulfurization reaction. The synthesized MOFs were characterized and analyzed by X-ray powder diffraction (XRD), Fourier transform infrared (FTIR), proton nuclear magnetic resonance (^1^H NMR), scanning electron microscopy (SEM), thermogravimetric analysis (TGA), nitrogen adsorption-desorption analysis, and microwave plasma-atomic emission spectrometer (MP-AES). The effect of individual and interaction of process variables were completely evaluated, which led to achieving the optimum values for all the MOFs in the ODS reaction. A polynomial quadratic model was designed to fully visualize the significance of the reaction temperature, oxidant agent mass ratio, and MOF amount, from which the response surface methodology (RSM) was adopted to determine the optimum values.

## 2 Material and Method

### 2.1 Material

Zirconium(IV) chloride (ZrCl_/_, 98%, Acros), terephthalic acid (99%, Acros), 2-hydroxyterephthalic acid (99%, Acros), nitroterephthalic acid (99%, Acros), hydrochloric acid (HCl, 36%, Honeywell), *N,N*-dimethylformamide (DMF, 99.5%, Fisher), ethanol (C_2_H_5_OH, 99.9%, Honeywell), acetonitrile (99.9%, Honeywell), *n*-dodecane (99%, Alfa Aesar), hydrogen peroxide (H_2_O_2_, 30%, Alfa Aesar), dibenzothiophene (98%, Acros), 2-methylbenzothiazole (99%, Acros), and 4,6-dimethyldibenzothiophene (97%, Alfa Aesar) were used without any further purification.

### 2.2 MOF Synthesis

MOFs were synthesized as originally described.^36^ Shortly, 1 g of ZrCl_4_ and the respective ligand (0.98 g terephthalic acid (BDC), 1.08 g 2-hydroxyterephthalic acid, and 1.25 g nitroterephthalic acid for UiO-66, UiO-66-OH, and UiO-66-NO_2_, respectively) were dissolved in 120 mL DMF and 8 mL HCl 36% and then sonicated for 35 min. The prepared solution was heated at 80 °C in an oven for 24 h. After cooling to room temperature, the sample was washed with DMF and ethanol and dried by heating under a 600 mbar pressure at 80 °C on a rotary evaporator.

### 2.3 Characterization Methods

X-ray powder diffraction (XRD) results were recorded on a X’Pert3 Powder (Malvern Panalytical) with a detector Cu Kα radiation with wavelength λ= 0.154 nm. Fourier transform infrared (FT-IR) spectra were recorded of the functional moieties of the samples on Nicolet iS50 FTIR spectrometric analyzer. The attenuated total reflectance (ATR-FTIR) sampling methodology was utilized for the direct examination of solid samples of UiO-66 derivate MOFs. Scanning electron microscopy (SEM) images were measured on a Zeiss FEG-SEM Ultra-55. Thermogravimetric analysis (TGA) was carried out on a Mettler-Toledo TGA 1. An Agilent 4200 microwave plasma atomic emission spectrometer was utilized to measure the elemental ratios and purity of a sample using microwave plasma atomic emission spectroscopy (MP-AES). At a sample temperature of 25 °C, digested MOF samples were evaluated by proton NMR spectroscopy on a 500 MHz Agilent DD2 NMR spectrometer with a 5 mm ID PFG probe head. Nitrogen adsorption–desorption isotherms were measured at 87 K on a Quantachrome Autosorb-iq device after the samples were degassed at 408 K for 18 h under vacuum, and then, specific surface areas were calculated using Quantachrome AsiQwin 5.23 software and BET method.

### 2.4 Catalyst Test

ODS reactions were conducted in a 22 mL reactor. For the model fuel (MF), 1000 ppm of each sulfur compound (DBT, 2-MB, and 4,6-DMDBT) was dissolved in *n*-dodecane. To this solution in the reactor, acetonitrile (3 mL) was added as the polar phase in a 1:1 ratio by volume. According to the experimental design, the specified amount of MOF was added to the reactor; hydrogen peroxide was supplied after the reactor was heated to a predefined temperature. The polar and fuel phases were separated after the reaction had completed and then analyzed by a Shimadzu QP2010 plus GC to measure the sulfur compounds. The sulfur removal efficiency was calculated according to eq 1:
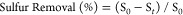
1

where S_0_ and S_*t*_ correspond to the sulfur concentrations in MF at the beginning and end of the reaction, respectively.

### 2.5 Design of Experiments

The oxidative desulfurization efficiency was investigated with the response surface methodology employing the central composite design (CCD).^37^ The parameters assessed were the reaction temperature (A), oxidative agent to sulfur mass ratio (B), MOF mass dosage (C), and selected MOF (D). The first three variables are designated at five levels as the numerical parameters and the fourth variable as a categorical parameter. The analysis of variance (ANOVA) was performed to evaluate the factors for the fitted quadratic model, and the fitness of the polynomial model was evaluated by the coefficient of determination R^2^.^38^ The F-test assessed the model and parameter significance, and the p-value with a specified confidence level was used to verify the model terms. The second-order polynomial equation is given in eq 2:
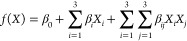
2

*X*_*i*_ and *X*_*j*_ are the coded values of variables, and β_0_, *β*_*i*_, and *β*_*ij*_ are the constant, linear, and interaction coefficients. Referring to the central composite design, 17 experimental runs were carried out including axial, factorial, and central runs. The oxidative sulfur removal reactions were conducted for each UiO-66 MOF. Table 2 summarizes the work domain classes for four parameters.

**Table 2:** Independent Test Variables at Five Levels Were Used in the Central Composite Design for Three Different MOFs, UiO-66(Zr), UiO-66-OH, and UiO-66-NO_2_^a^

			coded variable levels				
parameter	code	units	(-α)	(−1)	(0)	(+1)	(+α)
temperature	X_1_	°C	20	36.21	60	83.78	100
oxd./sul. mass ratio	X_2_	(mg/mg)	0.5	1.61	3.25	4.89	6
cat./sul. mass ratio	X_3_	(mg/mg)	0.5	3.44	7.75	12.06	15

a UiO-66: X_4_[0], UiO-66-OH: X_4_[1] , UiO-66-NO2: X_4_[2]α: 1.68

## 3 Results and Discussion

### 3.1 Characterization

**Figure 1:**
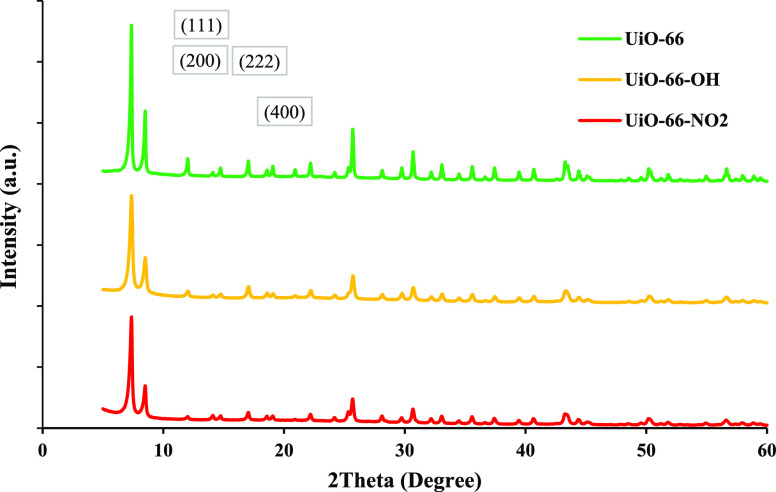
XRD powder patterns of UiO-66, UiO-66-OH, and UiO-66-NO_2_.

**Figure 2:**
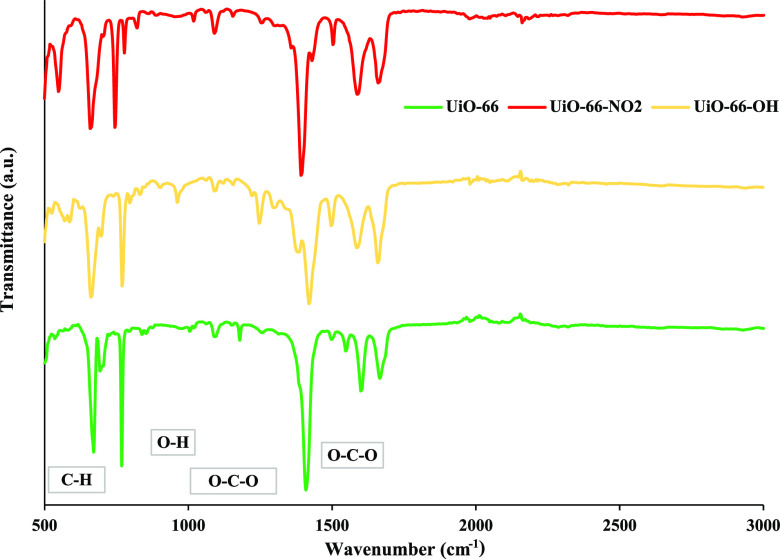
FT-IR spectrum of UiO-66, UiO-66-OH, and UiO-66-NO_2_.

Figure 1 shows how XRD was employed to assess the crystallinity and structure of the MOFs. The sample diffraction peaks of (111), (200), (022), (222), (400), (115), and (006) in the XRD patterns and indicates that the UiO-66, UiO-66-OH, and UiO-66-NO_2_ had been synthesized successfully.^39^ Synthesized UiO-66 derivatives were analyzed by FTIR, as shown in Figure 2. The IR bands for the three MOFs are in the same position at 660 cm^–1^ (C–H vibrations in BDC aromatic ring), 766 cm^–1^ (O–H vibrations in BDC aromatic ring), 1400 cm^–1^ (O–C–O symmetric stretch), and 1570 cm^–1^ (O–C–O asymmetric stretch).^40,41^

**Figure 3:**
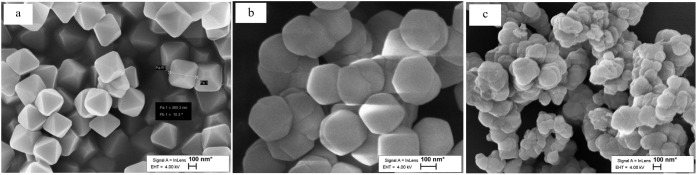
Typical SEM images of (a) UiO-66, (b) UiO-66-OH, and (c) UiO-66-NO_2_.

As illustrated in Figure 3, the SEM picture of the UiO-66 MOF exhibits a uniform octagonal shape. Based on the images, the particle sizes generally converged between 350 and 360 nm (Figure 3a). However, UiO-66-OH and UiO-66-NO_2_ demonstrated a higher irregularity of octagonal morphology with a crystal size between 340 and 370 nm (Figure 3b,c).

**Figure 4:**
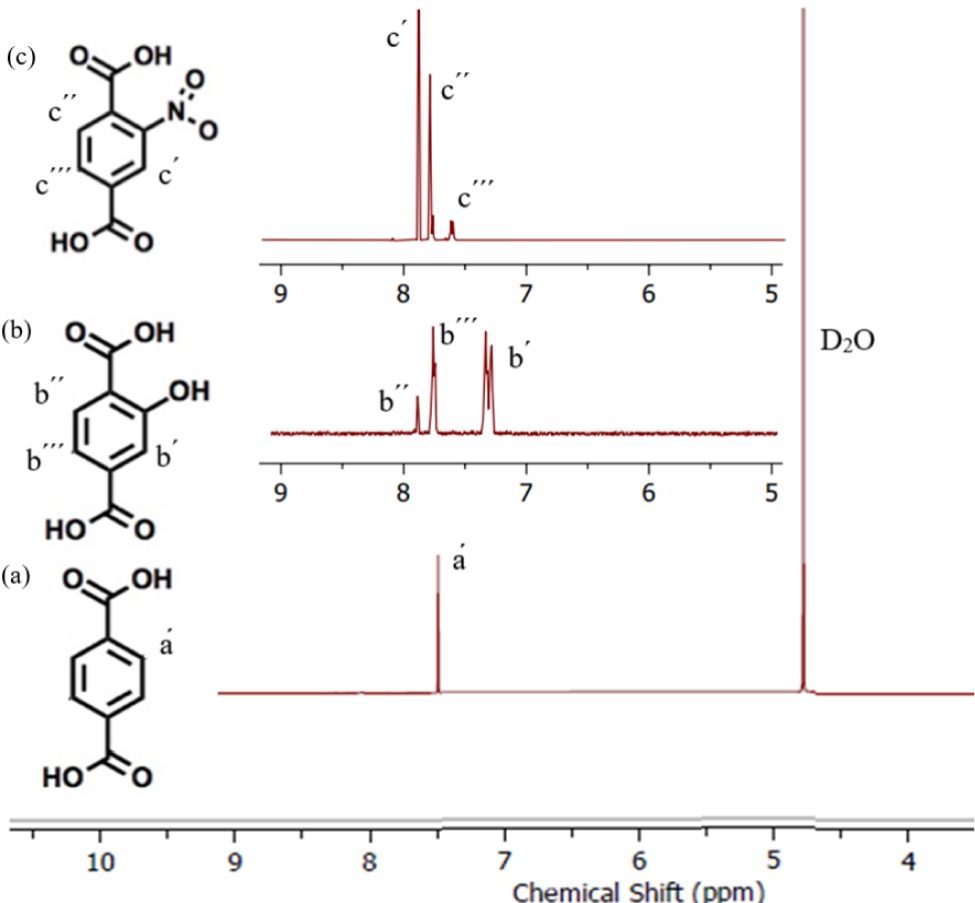
^1^H NMR spectra of the prepared (a) UiO-66, (b) UiO-66-OH, and (c) UiO-66-NO_2_ (NMR analyzed the bulk purity of solution after adding 650 uL of D_2_O to the digested solution of MOF sample in 10 drops of NaOD).

Figure 4 demonstrates the ^1^H NMR characterization of the prepared UiO-66 after digestion with NaOD/D_2_O. The peaks of UiO-66 show a signal at 7.48 ppm attributed to the BDC structure of the ligands.^42^ NMR analysis of the other functionalized UiO-66 displays three signals related to the hydroxyterephthalic acid and nitroterephthalic acid linkers in UiO-66-OH and UiO-66-NO_2_, respectively. The integrations of the NMR peaks for UiO-66 are illustrated in Figure S1.

**Figure 5:**
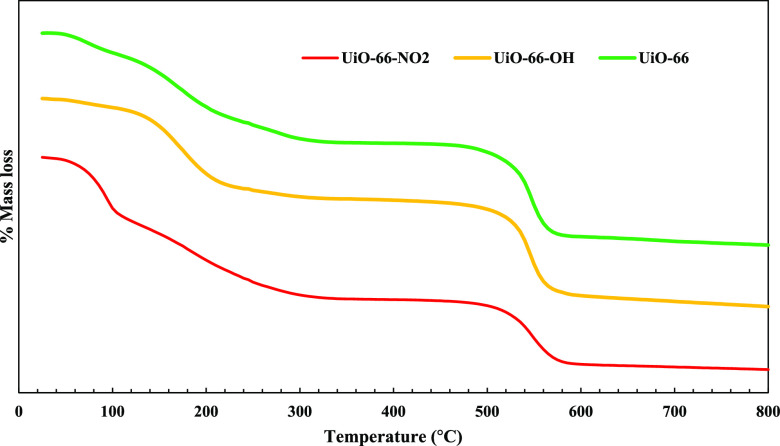
Thermogravimetry Curves of UiO-66, UiO-66-OH, and UiO-66-NO_2_. First mass loss (<100 °C), second mass loss (100–520 °C), third mass loss (>520 °C), UiO-66:16.9%, 27.11%, and 20.8%, UiO-66-OH: 3.2%, 29.3%, and 31.0%, UiO-66-NO_2_: 6.5%, 27.9%, and 30.4%, respectively.

Thermogravimetry analysis of UiO-66 derivates in Figure 5 shows a weight loss at 45–100 °C attributed to removing water and ethanol, and the other mass loss was related to eliminating *N,N*-dimethylformamide. At 520 °C, structure decomposition and (Zr) oxo-clusters dehydroxylation were the reasons for final mass loss.^43^Table 3 indicates the summarized isotherm data and the amount of zirconium in MOFs, as measured by MP-AES. BET surface area of UiO-66 is 1649 m^2^/g higher than UiO-66-OH and UiO-66-NO_2_ (852 m^2^/g and 776 m^2^/g). Additionally, UiO-66-NO_2_ observed a higher pore width of about 1.63 nm. Figure S2 summarizes the isotherm data for UiO-66 pristine and its derivatives.

**Table 3:** Zirconium Content and Textural Properties of Synthesized MOFs

MOF	amount of zirconium (wt %)	BET surface area (m^2^/g)	total pore volume (cc/g)	pore width (nm)
UiO-66	26.2	1649	0.605	1.11
UiO-66-OH	25.1	852	0.286	1.38
UiO-66-NO_2_	24.2	776	0.429	1.63

### 3.2 Effect of Parameters

Based on the Response Surface Methodology, experiments were conducted according to the given experimental design (Table 2). For UiO-66, UiO-66-OH, and UiO-66-NO_2_, the results of the defined factors, along with reaction temperature, oxidant over sulfur (Oxd./Sul. “mg/mg”), and MOF dosage over sulfur (cat./sul, mg/mg), were assessed at the given process time. As a result, Table S1 includes parameters, obtained values for the model, and experimental results. Three polynomial equations with coded factors for dibenzothiophene (DBT), 2-methylbenzothiazole (2-MB), and 4,6-dimethyldibenzothiophene (4,6-DMDBT) were derived by using multiple regression analysis to the experimental responses, as indicated in eqs 3, 4, and 5, respectively.
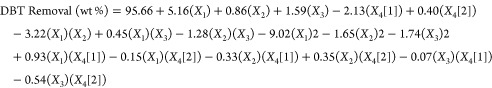
3
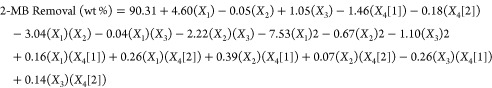
4
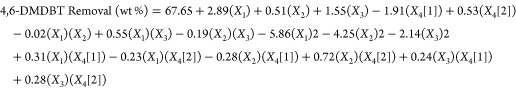
5

F-values and coefficient of determination (R^2^) were assessed by multiple regression and polynomial model fitness, respectively. The mathematical quadratic model was fitted by ANOVA to calculate the efficiency of sulfur removal. F-values of 18.24, 19.81, and 66.42 were obtained for 2-methylbenzothiazole (2-MB), dibenzothiophene (DBT), and 4,6-dimethyldibenzothiophene (4,6-DMDBT), respectively, as summarized in Table S2, and imply that for all cases, there are less than 0.01% chances that the particular F-value could occur due to noise. Additionally, a P-value lower than 0.05 demonstrated that experimental design models for sulfur removals are statistically significant. The coefficient of determination values confirmed that the model is successfully fitted to the data. (R^2^ values are 0.904, 0.911, and 0.921 for 2-MB, DBT, and 4,6-DMDBT, respectively).

**Figure 6:**
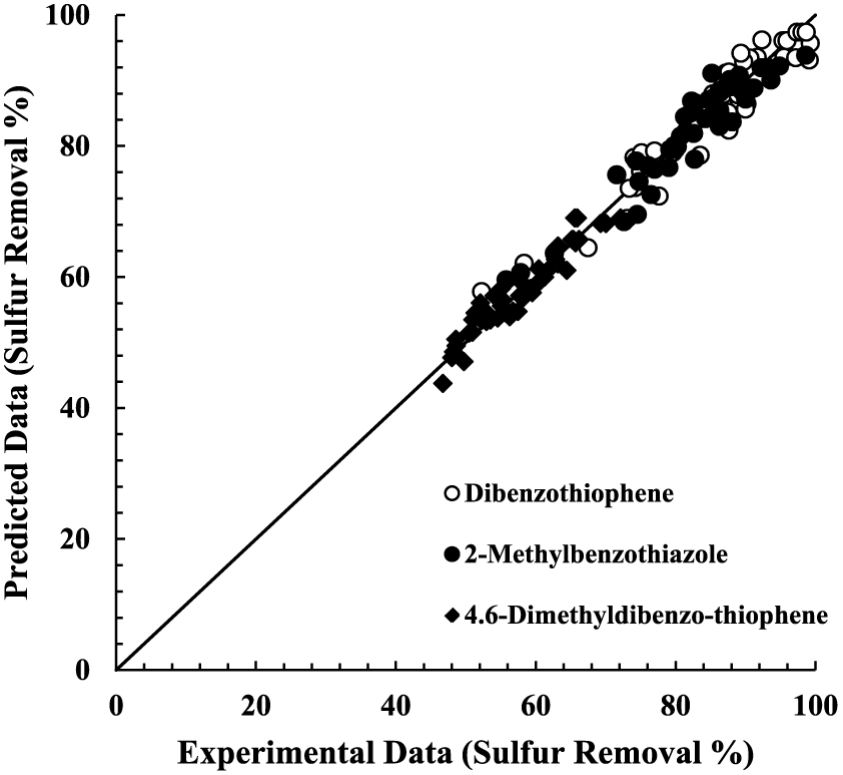
Comparison between model predicted and experimental values of sulfur removal efficiency.

The comparison between experimental and model values is shown in Figure 6; the curve demonstrates that model-predicted values are highly correlated with the obtained responses in the sulfur elimination yield. The root-mean-square deviation of the experimental results and predicted model was 3.4%.

**Figure 7:**
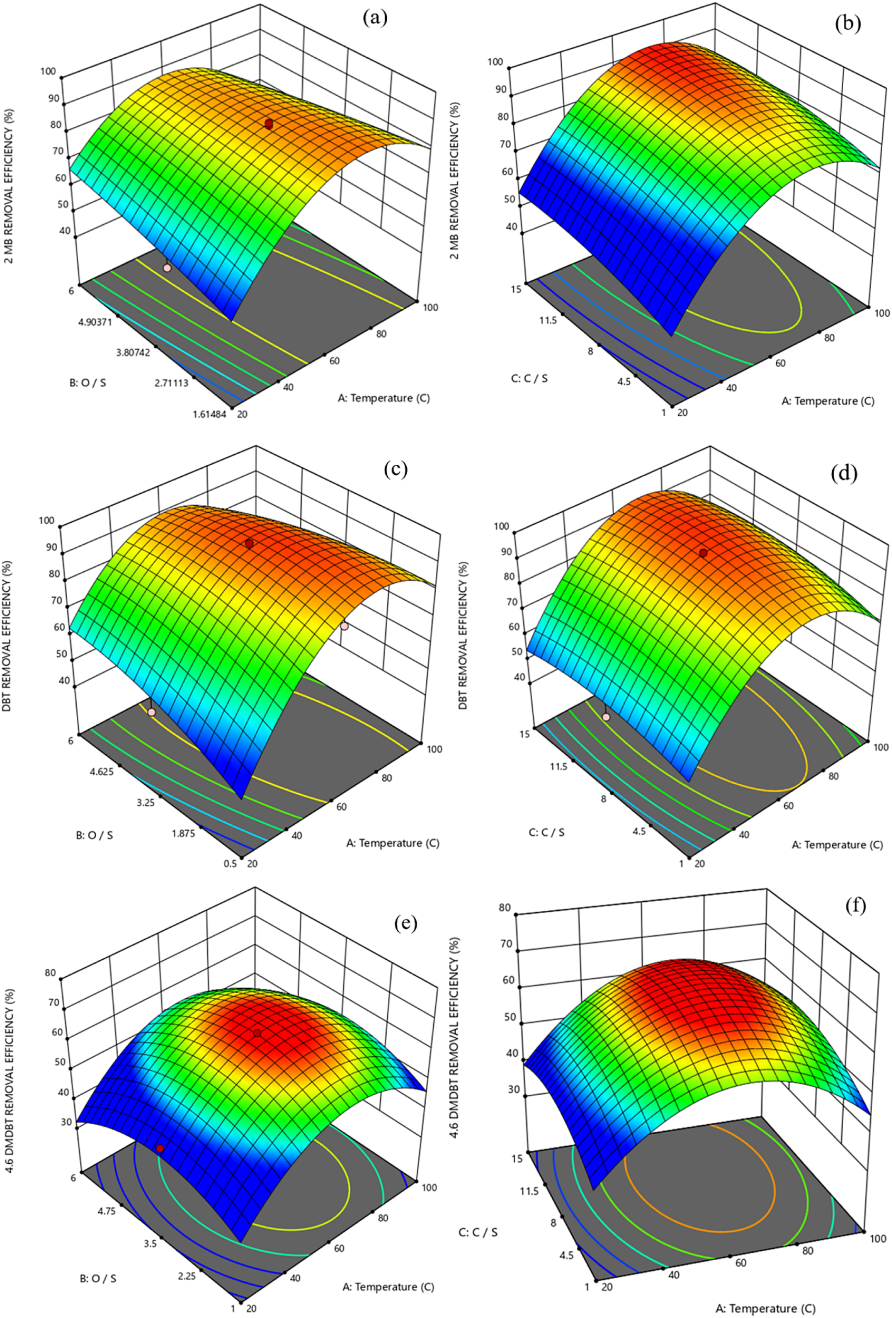
3D plot presenting the performance of UiO-66 MOF on the removal of sulfur from the MF. (a) Sulfur removal efficiency: 2-MB. Constant parameter: Cat./Sul. mass ratio (X_3_). Constant parameter value (mg/mg): 9.8. (b) Sulfur removal efficiency: 2-MB. Constant parameter: Oxd./Sul. mass ratio (X_2_). Constant parameter value (mg/mg): 2.9. (c) Sulfur removal efficiency: DBT. Constant parameter: Cat./Sul. mass ratio (X_3_). Constant parameter value (mg/mg): 9.8. (d) Sulfur removal efficiency: DBT. Constant parameter: Oxd./Sul. mass ratio (X_2_). Constant parameter value (mg/mg): 2.9. (e) Sulfur removal efficiency: 4,6-DMDBT. Constant parameter: Cat./Sul. mass ratio (X_3_). Constant parameter value (mg/mg): 9.8. (f) Sulfur removal efficiency: 4,6-DMDBT. Constant parameter: Oxd./Sul. mass ratio (X_2_). Constant parameter value (mg/mg): 2.9.

The normal plot of the residuals in Figure S3 demonstrates the reliability of the model. The straight lines for DBT, 2-MB, and 4,6-DMDBT indicate that the residuals have a normal distribution. This study was carried out to determine the impact of individual factors and their interactions by employing the response surface methodology. The effect of reaction temperature, oxd./sul. ratio, and cat./sul. ratio for UiO-66, UiO-66-OH, and UiO-66-NO_2_ on the efficiency of sulfur removal are displayed in 3D surface plots (Figures 7, S4, and S5, respectively). Figure 7a shows the effect of temperature and oxd./sul. on the ODS of 2-MB for UiO-66. The highest yield of 2-MB oxidation (89.96%) was obtained at the oxd./sul. mass ratio of 1.5:1.7 and a temperature range of 70–73 °C. Figure 7b shows 3D plots between the temperature and MOF dosage, illustrating that two factors have a significant impact on 2-MB oxidation. The results indicated that in the temperature range between 60 and 70 °C, the higher catalyst to sulfur mass ratio between 11.5 and 12.5 was in favor of 2-MB ODS efficiency.

**Figure 8:**
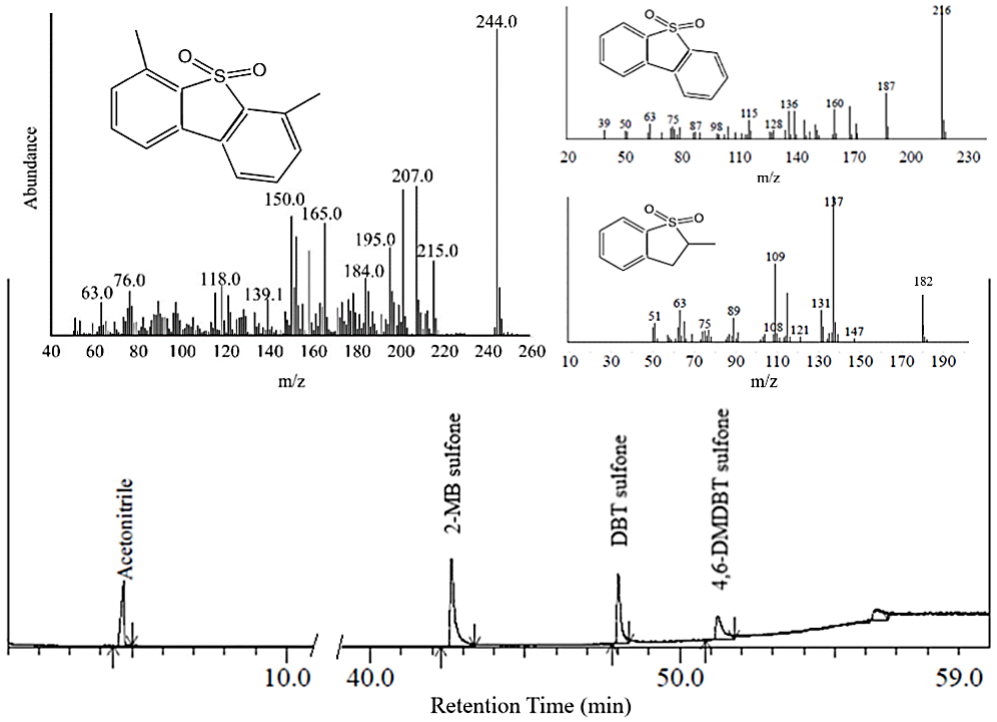
MS spectra of the oxidized products of 2-MB (*m*/*z*: 182), DBT (*m*/*z*: 216), and 4,6-DMDBT (*m*/*z*: 244).

Figure 8c,d demonstrate the effect of oxd./sul. and cat./sul. ratio versus temperature for the DBT ODS reaction. It shows that the three parameters have visible effects on oxidation. For the case of interaction between reaction temperature and oxd./sul. ratio (Figure 7c), at the oxd./sul. ratio of 0.5:2.3 and a temperature of 71 °C, the maximum efficiency of 4,6-dimethyldibenzothiophene removal was observed (95%). Furthermore, the calculated optimal amount of cat./sul. was 10.8 (mg/mg) for the highest DBT removal efficiency (Figure 7d).

At a particular oxidant over sulfur ratio range of 1.0:3.0, as shown in Figure 7e, an increased 4,6-DMDBT oxidation efficiency was observed when the temperature was raised from 65 to 72 °C. However, the removal yield of 4,6-DMDBT declined with temperature increase over 73 °C. Because the oxidative desulfurization process is endothermic,^44^ raising the temperature promotes the sulfur removal rate by increasing the molecular mobility of reaction components. In contrast, at higher temperatures, the rate of oxygen peroxide decomposition also increases, and as a result, the oxidation reaction rate falls due to the oxidant concentration decrease.^45^ When the oxd./sul. mass ratio grows from 2.4 to 3.3 at a specified temperature, the 4,6-DMDBT removal rate increases and then starts to decrease as the oxd./sul. ratio rises to 6 (Figure 7e). On the contrary, the removal of sulfur was more efficient if there was a higher amount of oxidant.^46,47^

According to the ANOVA analysis, oxd./sul = 3.0 was the optimum value, and Figure 7f illustrates the interaction between reaction temperature and cat./sul on the 4,6-DMDBT removal yield. Perceptibly, the highest sulfur removal efficiency (about 64%) was achieved at the temperature of 60–75 °C and cat./sul of 9.8–10.5. However, further rising cat./sul to 15, the removal efficiency is decreased because catalytic activity of the excessive MOF in the oxidation process is much lower due to aggregation.^48,49^

Additionally, Figures S4 and S5 illustrate the 3D surface response graphs for UiO-66-OH and UiO-66-NO_2_, respectively. As shown, UiO-66-NO_2_ achieved higher sulfur removing (for three sulfur planar compounds) in 150 min of reaction time, and after UiO-66-OH, UiO-66, and UiO-66-NH_2_,^31^ respectively.

The prominent parameters in the desulfurization process are the electron density of the sulfur atoms and the steric hindrance surrounding the sulfur atoms. Oxidative desulfurization benefits from a greater electron density and a lower steric hindrance. Sulfur atoms in 4,6-dimethyldibenzothiophene, dibenzothiophene, and 2-methylbenzothiazole have electron densities of 5.760, 5.758, and 5.739, respectively.^50^

Because the electron densities on the sulfur atoms of 4,6-dimethyldibenzothiophene and dibenzothiophene are comparable, the steric hindrance surrounding the sulfur atom had a major role in the oxidation. As a result, dibenzothiophene indicated a larger conversion for derivatives of UiO-66 because there was less steric hindrance around the sulfur atom among the planar compounds. The low activity for 4,6-dimethyldibenzothiophene is due to the larger size, than DBT and 2-MB, which could mostly interact with the external surface of the MOFs.^51^ Thus, the activity with UiO-66 and its derivatives followed the sequence DBT > 2-MB > 4,6-DMDBT.

### 3.3 Parameter Optimization

The optimum reaction conditions for the removal of sulfur-containing compounds from MF have been obtained using the response surface methodology in the CCD model. The optimal values of three parameters are summarized in Table 4 for achieving the maximum sulfur removal as well as the model desirability.

**Table 4:** Obtained Optimal Values for UiO-66, UiO-66-OH, and UiO-66-NO_2_ in Achieving Highest Sulfur Removal, Standard Uncertainties (u) are u (T) = ± 1 °C; u (Oxd./Sul. Mass Ratio) = ± 0.01; u (Oxd./Sul. Mass Ratio) = ± 0.01 wt %; u (Yield) = ± 0.01 wt %

	optimum value						
catalyst	temperature (°C)	oxd./sul.ratio (−)	cat./sul.ratio (−)	2-MB removal (%)	DBT removal (%)	4,6-DMDBT removal (%)	desirability
UiO-66	67.9	3.02	9.84	89.96	95.09	66.51	0.828
UiO-66-OH	66.8	3.21	9.71	91.26	96.94	68.90	0.883
UiO-66-NO_2_	66.7	2.97	9.75	93.23	98.68	69.33	0.916

The impact of UiO-66 on DBT removal efficiency is summarized in Table 5. Regarding the extraction and oxidation (without MOF), the results indicated that the removal of sulfur is significantly less efficient compared to the runs with catalysts when maintaining the oxd./sul. ratio at 3.0 in four different temperature runs. Compared to the previous study,^30^ highest DBT removal efficiency achieved was 89.7% when UiO-66-NH_2_ (in the conditions of 72.6 °C temperature, 1.62 O/S mass ratio mg/mg, and 12.1 C/S mass ratio mg/mg) was used as the catalyst, which is lower than the yields in the case of UiO-66.

**Table 5:** Catalytic Oxidative and Extractive Removal of Dibenzothiophene with and without UiO-66 in the Presence of H_2_O_2_ from the Model Fuel

	DBT removal at different temperatures (%)			
	36 °C	60 °C	68 °C	84 °C
UiO-66	75%	94%	95%	91%
No catalyst^a^	25%	32%	34%	34%

a Reaction condition: 3 mL of MF, 13.1 μL of H_2_O_2_ (oxd./sul. = 3.0), 3 mL of polar phase, for 150 min.

Upon the oxidative process and separation of the polar phase, the oxidation product of 2-MB, DBT, and 4,6-DMDBT were analyzed by gas chromatography, as shown in Figure 8. From the analysis results, the sulfones of 2-MB, DBT, and 4,6-DMDBT have been detected.

### 3.4 Reusability of MOF

**Figure 9:**
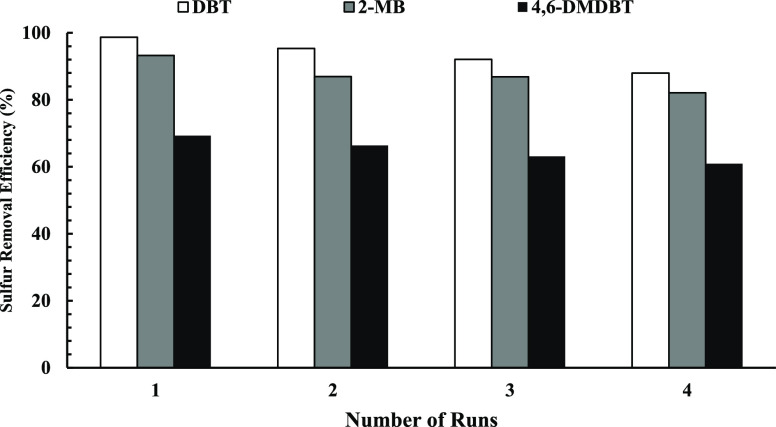
Sulfur removal yield on UiO-66-NO_2_ catalyst in four cycles (3 mL of MF, 62 mg of catalyst (cat./sul.= 9.8), 13.1 μL of H_2_O_2_ (oxd./sul. = 3.0), 3 mL of polar phase, 150 min, at 67 °C).

The recovery of the MOF is a factor in industrial utilization and economic issues. Reusability of the prepared MOFs was investigated by conducting four catalytic oxidation tests at 67 °C, cat./sul.= 9.8, and oxd./sul. = 3.0 in the optimum point. Centrifugation was utilized to separate the catalyst from the liquid phase and recover it following each experiment. The separated catalyst was washed with acetonitrile and dried at 100 °C for 12 h in the oven at atmospheric pressure and then employed in the following catalytic oxidation reaction. Sulfur removal efficiency for four multiple reactions demonstrates (Figure 9) that with a nearly 10.8% decrease, the initial removal yield was retained, descending from 98.7 to 93.3%, 69.3 to 88.0%, and 82.1 to 61.0% for DBT, 2-MB, and 4,6-DMDBT, respectively. The deactivation mechanism of UiO-66 active sites for sequential cycles has been previously reported.^30,52^ The reusability of UiO-66-OH and UiO-66 MOFs is shown in Figure S6.

## 4 Conclusion

Metal Organic Framework UiO-66 and its derivatives were prepared by solvothermal synthesis. The prepared catalysts were characterized by FTIR, proton NMR, XRD, SEM, TGA, BET, and MP-AES analyses. Response surface methodology and central composite design were employed to measure the impact of reaction conditions, temperature (X_1_), oxidant mass ratio (X_2_), MOF mass ratio (X_3_), and UiO-66-x (X_4_) on ODS efficiency. The obtained F-value and coefficient of determination (R^2^) implied that experimental and predicted results by the model were fitted at an acceptable level. The sulfur removal efficiency could reach >95%, > 90%, and >67% of DBT, 2-MB and 4,6-DMDBT, respectively, in 66–68 °C, oxd./sul. ratio: 3.0–3.2, and cat./sul. ratio: 9.7–9.8 for MF (1000 ppm each sulfur content) for three employed MOFs. UiO-66-NO_2_ exhibited higher catalytic activity in the deep oxidative desulfurization due to the presence of more electron-accepting group in its ligand structure. MOFs exhibited high reusability and maintained their activity over multiple runs in the oxidation of planar sulfur compounds.

## Supporting Information Available

Fundamental characterizations and analysis result of response surface methodology (Table S1); analysis of variance for the quadratic model of sulfur planal removal efficiency (Table S2); ^1^H-NMR spectrum of pristine UiO-66 (Integrations of the NMR peaks) (Figure S1); N_2_ adsorption isotherms by the BET analysis of the UiO-66 derivate composite (Figure S2); externally studentized residuals versus normal probability of the model for (a) DBT, (b) 2-MB, and (c) 4,6-DMDBT removal efficiency (Figure S3); 3D plot presenting the performance of UiO-66-OH MOF on the removal of sulfur from the MF (Figure S4) (PDF)
