# Supplementary material for: Deep Oxidative Desulfurization of Planar Compounds Over Functionalized Metal–Organic Framework UiO-66(Zr): An Optimization Study
Source: ACS Omega. 2024 May 23;9(22):23329–38. doi: 10.1021/acsomega.3c09971 (PMC11154902; doi:10.1021/acsomega.3c09971)
Supplement: Supplementary file 1 — ao3c09971_si_001.pdf [file ao3c09971_si_001.pdf]

Supporting Information

Deep Oxidative Desulfurization of Planar Compounds over Functionalized Metal Organic Framework UiO-66(Zr): An Optimization Study

Bijan Barghi[*(a)], *Tanel Mõistlik* [(a)], *Anastassia Raag* [(a)], *Maria Volokhova* [(b)], *Indrek Reile* [(b)], *Liis Seinberg* [(b)], *Valdek Mikli* [(c)], *Allan Niidu* [(a)]

(a) Virumaa College School of Engineering, Tallinn University of Technology, Järveküla 75, 30322 Kohtla-Järve, Estonia

(b) National Institute of Chemical Physics and Biophysics, Akadeemia 23, 12618 Tallinn, Estonia

(c) Department of Materials and Environmental Technology, Tallinn University of Technology, 19086 Tallinn, Estonia

[*] *Corresponding author: bijan.barghi@taltech.ee*

Table S1. Response Surface Methodology arrangement, design matrix for predicted model and experimental data

Test Run No.	Order Type	Catalyst	X_1	X_2	X_3	2-MB removal efficiency (%)		DBT removal efficiency (%)		4,6-DMDBT removal efficiency (%)	
			(˚C)	(-)	(-)	Exp.	Pred.	Exp.	Pred.	Exp.	Pred.
1	Axial	UiO-66-OH	83.78	1.61	3.44	86.27	85.48	86.86	87.90	56.72	54.80
2	Center	UiO-66-OH	60.00	3.25	7.75	88.08	90.14	95.60	96.07	70.02	68.18
3	Factorial	UiO-66-OH	60.00	3.25	0.50	82.09	85.02	87.95	89.38	58.03	59.05
4	Axial	UiO-66-OH	36.22	4.89	3.44	79.86	79.93	82.85	83.75	53.51	53.44
5	Factorial	UiO-66-OH	100.00	3.25	7.75	76.07	77.04	75.10	78.97	52.09	56.08
6	Axial	UiO-66	83.78	4.89	3.44	86.26	83.00	87.73	83.84	57.45	54.75
7	Center	UiO-66	60.00	3.25	7.75	91.18	88.85	95.65	93.53	66.18	65.74
8	Center	UiO-66-NO_2	60.00	3.25	7.75	92.56	91.95	98.68	97.39	65.57	69.02
9	Center	UiO-66-NO_2	60.00	3.25	7.75	93.24	91.95	98.06	97.39	72.11	69.02
10	Factorial	UiO-66-NO_2	60.00	0.50	7.75	85.17	91.08	87.62	91.32	58.25	56.89
11	Axial	UiO-66-OH	36.22	1.61	3.44	74.49	69.56	77.61	72.34	48.55	50.53
12	Axial	UiO-66-OH	83.78	4.89	12.06	80.70	81.51	89.11	86.88	63.13	62.01
13	Factorial	UiO-66-NO_2	60.00	3.25	0.50	82.30	86.86	86.95	88.78	60.41	61.24
14	Axial	UiO-66-NO_2	36.22	4.89	12.06	80.25	79.90	87.55	85.12	53.18	54.34
15	Factorial	UiO-66	60.00	0.50	7.75	82.86	86.57	85.33	87.98	52.26	53.35
16	Factorial	UiO-66-NO_2	100.00	3.25	7.75	74.38	77.70	77.01	79.25	54.03	57.17
17	Axial	UiO-66-OH	83.78	4.89	3.44	88.08	83.66	90.17	86.44	59.49	57.62
18	Axial	UiO-66-NO_2	83.78	1.61	12.06	98.62	93.83	99.25	95.68	61.97	61.32

Test Run No.	Order Type	Catalyst	X_1	X_2	X_3	2-MB removal efficiency (%)		DBT removal efficiency (%)		4,6-DMDBT removal efficiency (%)	
			(˚C)	(-)	(-)	Exp.	Pred.	Exp.	Pred.	Exp.	Pred.
19	Axial	UiO-66-NO_2	83.78	1.61	3.44	90.01	87.14	88.90	87.82	58.98	57.75
20	Axial	UiO-66	83.78	1.61	3.44	84.31	84.18	85.78	86.66	56.27	53.95
21	Center	UiO-66	60.00	3.25	7.75	90.18	88.85	91.65	93.53	65.34	65.74
22	Axial	UiO-66-OH	36.22	4.89	12.06	82.70	77.95	87.61	82.39	55.08	55.60
23	Factorial	UiO-66-NO_2	60.00	3.25	15.00	89.06	90.79	92.33	96.17	63.16	64.72
24	Factorial	UiO-66	60.00	3.25	15.00	84.70	87.05	87.17	91.16	62.81	62.70
25	Axial	UiO-66	36.22	4.89	3.44	79.14	79.47	79.61	79.00	48.61	49.50
26	Axial	UiO-66-NO_2	36.22	1.61	12.06	79.94	79.46	79.85	79.56	55.64	54.55
27	Axial	UiO-66-OH	83.78	1.61	12.06	94.82	92.20	97.17	93.46	61.26	59.97
28	Factorial	UiO-66-OH	60.00	0.50	7.75	86.23	88.39	88.63	89.38	53.17	54.09
29	Axial	UiO-66-NO_2	36.22	1.61	3.44	76.48	72.60	73.38	73.51	52.94	53.19
30	Axial	UiO-66	36.22	4.89	12.06	79.03	76.68	83.50	78.57	50.96	51.58
31	Axial	UiO-66-NO2	83.78	4.89	12.06	81.41	82.09	89.51	88.36	64.45	61.04
32	Factorial	UiO-66-OH	60.00	6.00	7.75	86.31	88.12	90.62	93.44	55.07	58.25
33	Axial	UiO-66-NO_2	36.22	4.89	3.44	82.56	81.92	82.96	84.18	54.56	53.77
34	Factorial	UiO-66-NO_2	20.00	3.25	7.75	62.66	63.63	67.47	64.49	48.01	47.70
35	Factorial	UiO-66	20.00	3.25	7.75	55.77	59.56	52.24	57.77	46.71	43.77
36	Center	UiO-66-OH	60.00	3.25	7.75	87.75	90.14	95.95	96.07	70.05	68.18
37	Factorial	UiO-66	60.00	6.00	7.75	85.43	87.37	87.90	89.77	52.91	54.11
38	Axial	UiO-66	36.22	1.61	12.06	74.77	74.56	74.24	73.63	50.47	51.47
39	Axial	UiO-66	83.78	4.89	12.06	79.81	80.04	84.28	85.21	60.26	59.04

Test Run No.	Order Type	Catalyst	X_1	X_2	X_3	2-MB removal efficiency (%)		DBT removal efficiency (%)		4,6-DMDBT removal efficiency (%)	
			(˚C)	(-)	(-)	Exp.	Pred.	Exp.	Pred.	Exp.	Pred.
40	Factorial	UiO-66	100.00	3.25	7.75	71.60	75.58	74.07	78.25	51.32	54.53
41	Factorial	UiO-66-OH	60.00	3.25	15.00	87.86	89.01	89.72	92.91	65.68	65.22
42	Factorial	UiO-66	60.00	3.25	0.50	81.27	84.42	83.74	86.07	55.07	56.69
43	Center	UiO-66-NO_2	60.00	3.25	7.75	92.24	91.95	97.37	97.39	65.94	69.02
44	Center	UiO-66	60.00	3.25	7.75	91.02	88.85	95.49	93.53	65.17	65.74
45	Axial	UiO-66	83.78	1.61	12.06	93.63	90.09	99.10	93.14	60.27	59.02
46	Factorial	UiO-66-OH	20.00	3.25	7.75	57.83	60.67	58.34	62.11	49.73	47.11
47	Axial	UiO-66-OH	36.22	1.61	12.06	76.99	76.46	74.98	76.10	51.00	53.49
48	Factorial	UiO-66-NO_2	60.00	6.00	7.75	88.72	89.05	89.33	94.15	57.71	57.14
49	Axial	UiO-66-NO_2	83.78	4.89	3.44	86.59	84.28	89.96	85.62	59.24	58.25
50	Center	UiO-66-OH	60.00	3.25	7.75	89.28	90.14	95.36	96.07	69.23	68.18
51	Axial	UiO-66	36.22	1.61	3.44	72.62	68.47	73.09	68.94	48.25	48.61

Table S2. Analysis of Variance for the quadratic model of sulfur planal removal efficiency

Source	Degree of Freedom	2-MB efficiency (%)		DBT efficiency (%)		4,6-DMDBT efficiency (%)	
		F -Value	p-value	F -Value	p-value	F -Value	p-value
A: Temperature (℃)	1	79.81	< 0.0001	81.86	< 0.0001	2.08	0.1587
B: OXD./SUL.(mg/mg)	1	0.0842	0.7735	2.26	0.1420	19.11	0.0001
C: CAT./SUL.(mg/mg)	1	4.11	0.0507	7.73	0.0089	9.57	0.0005
D: MOF	2	3.77	0.0334	4.90	0.0137	0.0019	0.9652
AB	1	20.43	< 0.0001	18.62	0.0001	1.43	0.2410
AC	1	0.0042	0.9488	0.3634	0.5507	0.1999	0.8198
AD	2	0.1711	0.8435	0.7564	0.4773	0.1791	0.6749
BC	1	10.84	0.0024	2.94	0.0956	1.05	0.3603
BD	2	0.2266	0.7985	0.1173	0.8897	0.5297	0.5937
CD	2	0.0658	0.9364	0.3408	0.7137	225.12	< 0.0001
A^2	1	175.94	< 0.0001	206.40	< 0.0001	118.01	< 0.0001
B^2	1	1.38	0.2489	6.87	0.0131	29.88	< 0.0001
C^2	1	3.78	0.0603	7.67	0.0092	2.08	0.1587
Model	17	18.24	< 0.0001	19.81	< 0.0001	66.42	< 0.0001

Source	Degree of Freedom	2-MB efficiency (%)		DBT efficiency (%)		4,6-DMDBT efficiency (%)	
		F -Value	p-value	F -Value	p-value	F -Value	p-value
Lack of Fit	27	32.96	0.0001	8.45	0.0067	1.13	0.4803
R^2		0.904		0.911		0.921	
Adjusted R^2		0.854		0.865		0.880	

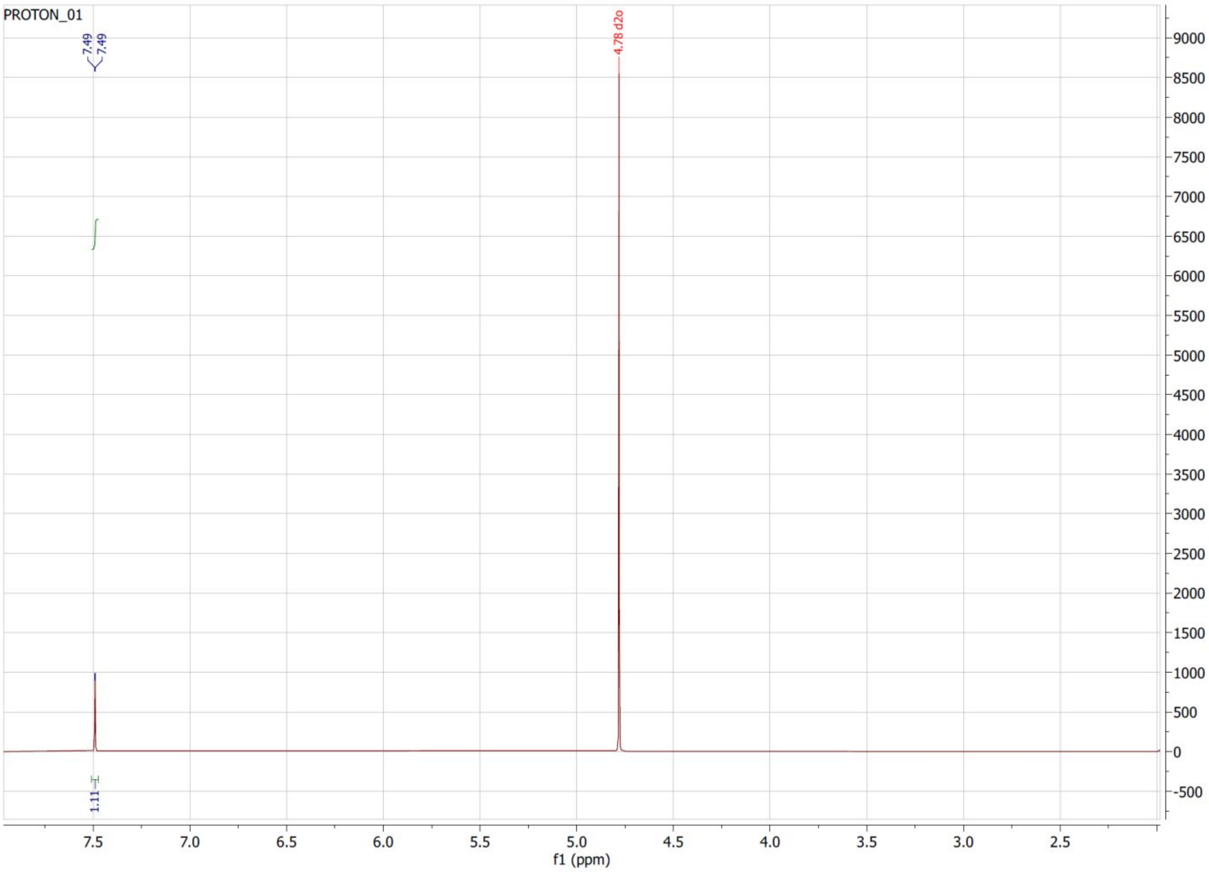

Figure S1. ^{1}H-NMR spectrum of pristine UiO-66 (Integrations of the NMR peaks)

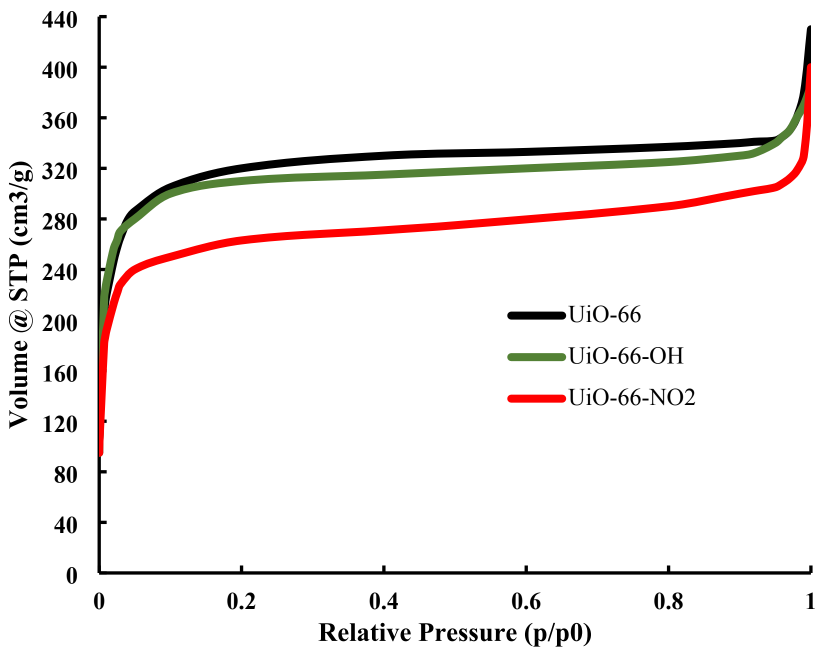

Figure S2. N_2 adsorption isotherms by the BET analysis of the UiO-66 derivates composite

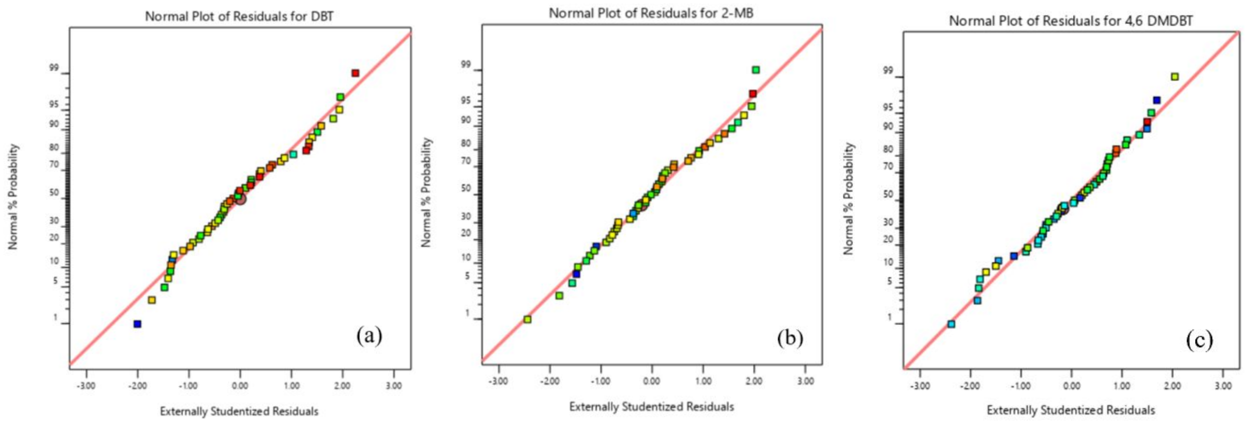

Figure S3. Externally studentized residuals versus Normal probability of the model for (a) DBT, (b) 2-MB, and (c) 4,6-DMDBT removal efficiency

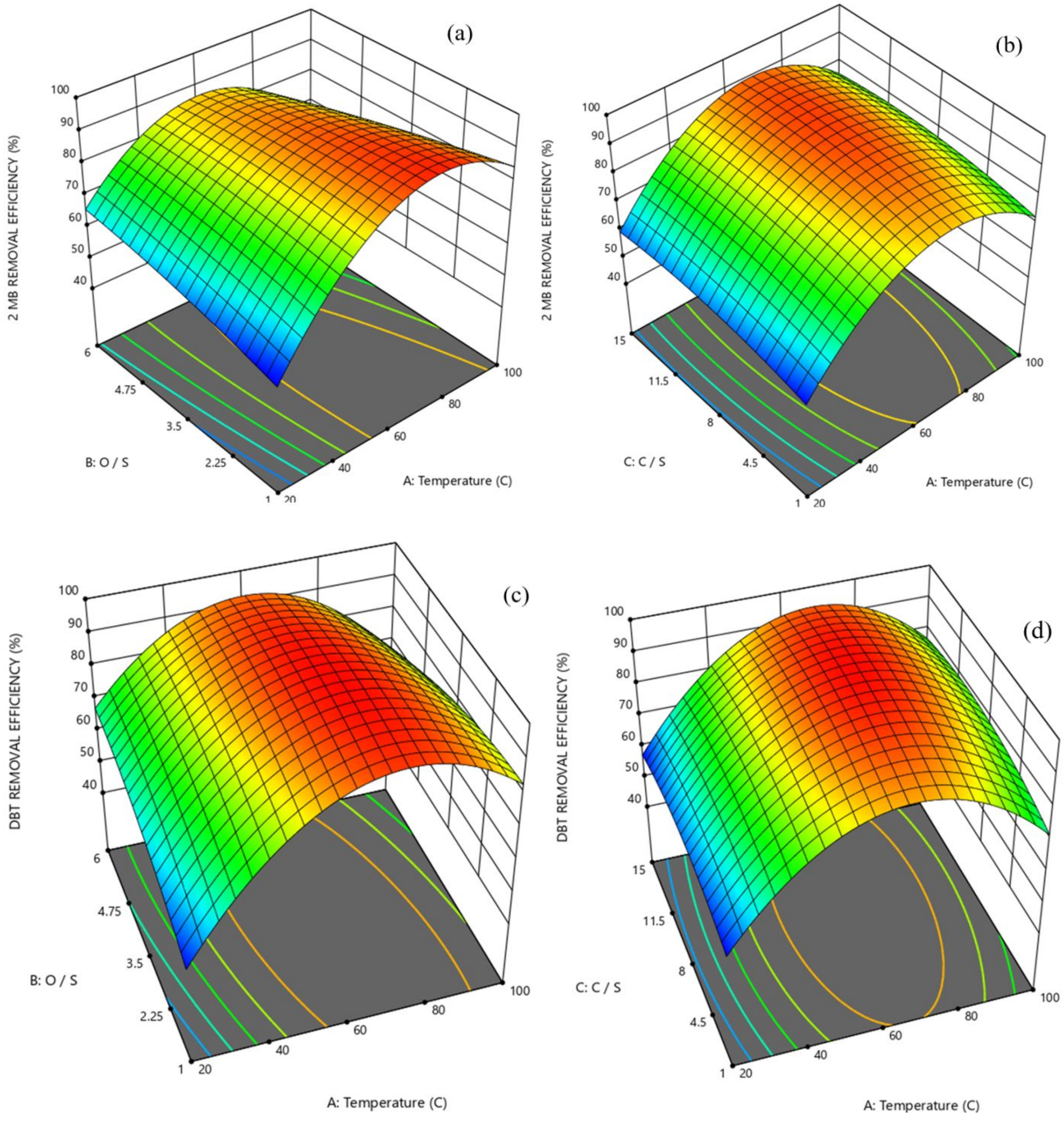
(a)
2 MB REMOVAL EFFICIENCY (%)
B: O / S
A: Temperature (C)
(b)
2 MB REMOVAL EFFICIENCY (%)
C: C / S
A: Temperature (C)
(c)
DBT REMOVAL EFFICIENCY (%)
B: O / S
A: Temperature (C)
(d)
DBT REMOVAL EFFICIENCY (%)
C: C / S
A: Temperature (C)

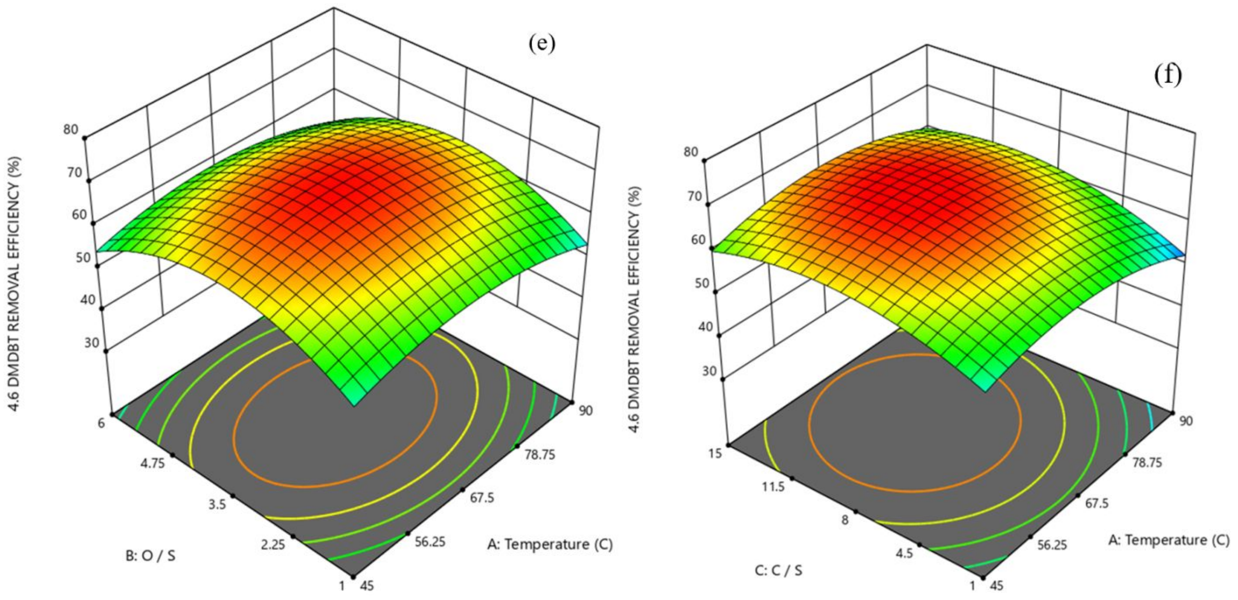

Figure S4. 3D plot presenting the performance of UiO-66-OH MOF on the removal of sulfur from the MF. The effect of parameters as:

Figure	Sulfur Removal Efficiency	Constant Parameter	Constant Parameter value (mg/mg)
(a)	2-MB	Cat./Sul. mass ratio (X_3)	9.7
(b)	2-MB	Oxd./Sul. mass ratio (X_2)	3.2
(c)	DBT	Cat./Sul. mass ratio (X_3)	9.7
(d)	DBT	Oxd./Sul. mass ratio (X_2)	3.2
(e)	4,6-DMDBT	Cat./Sul. mass ratio (X_3)	9.7
(f)	4,6-DMDBT	Oxd./Sul. mass ratio (X_2)	3.2

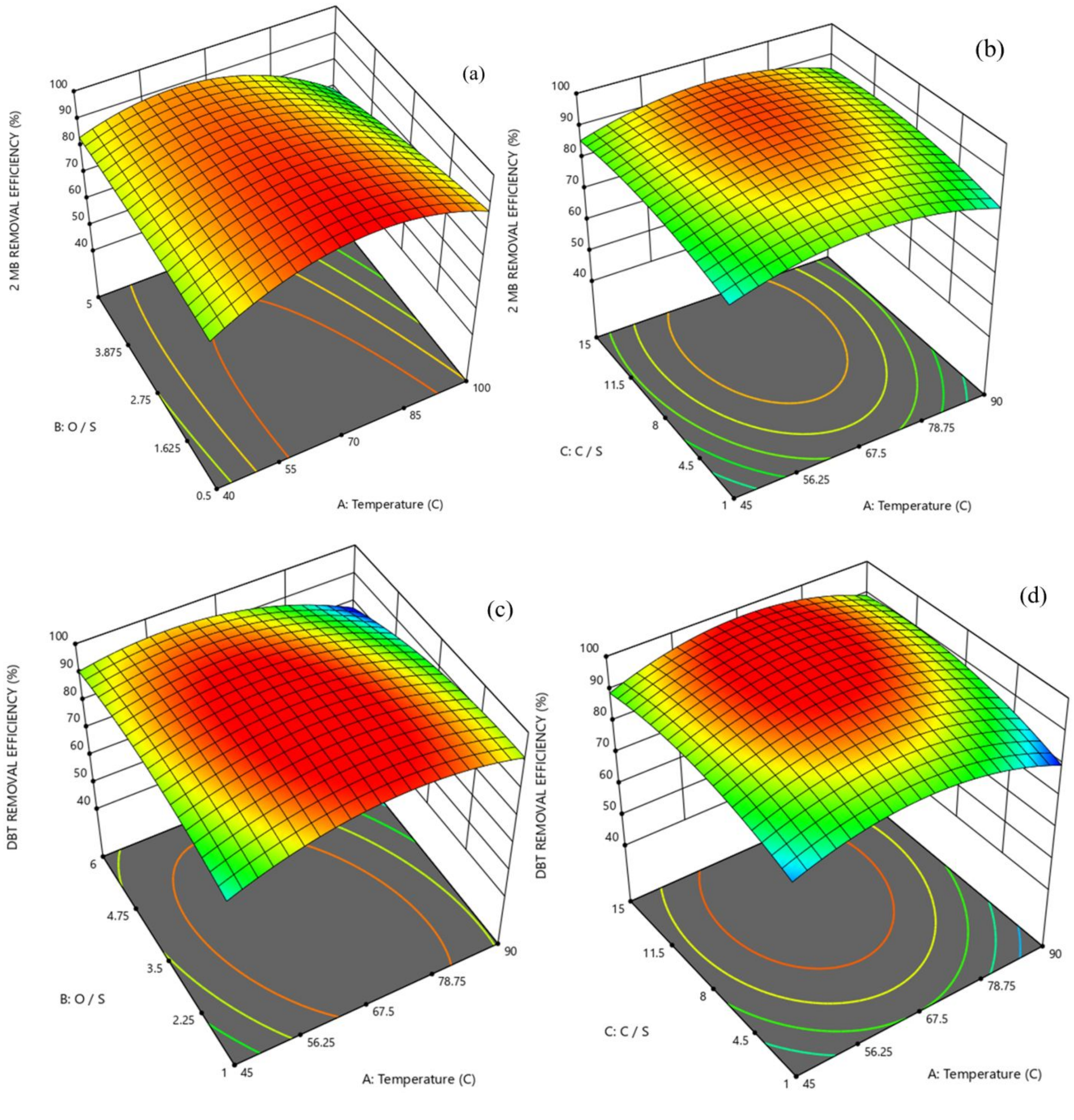
(a)
2 MB REMOVAL EFFICIENCY (%)
B: O / S
A: Temperature (C)
(b)
2 MB REMOVAL EFFICIENCY (%)
C: C / S
A: Temperature (C)
(c)
DBT REMOVAL EFFICIENCY (%)
B: O / S
A: Temperature (C)
(d)
DBT REMOVAL EFFICIENCY (%)
C: C / S
A: Temperature (C)

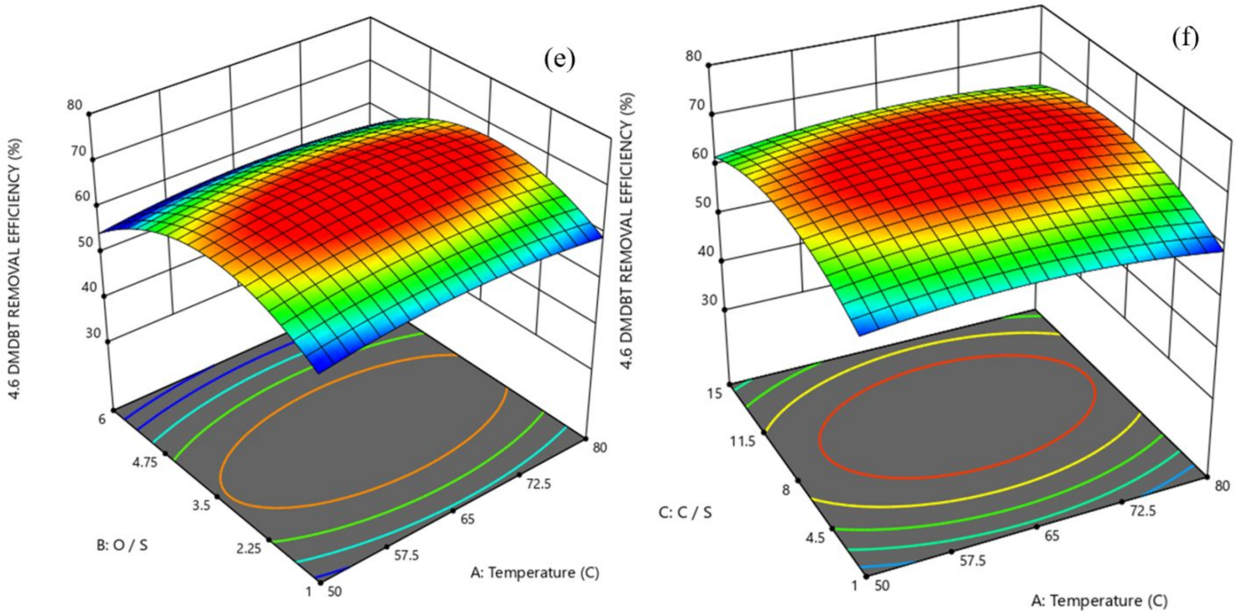

Figure S5. 3D plot presenting the performance of UiO-66-NO_2 MOF on the removal of sulfur from the MF. The effect of parameters as:

Figure	Sulfur Removal Efficiency	Constant Parameter	Constant Parameter value (mg/mg)
(a)	2-MB	Cat./Sul. mass ratio (X_3)	9.7
(b)	2-MB	Oxd./Sul. mass ratio (X_2)	3.0
(c)	DBT	Cat./Sul. mass ratio (X_3)	9.7
(d)	DBT	Oxd./Sul. mass ratio (X_2)	3.0
(e)	4,6-DMDBT	Cat./Sul. mass ratio (X_3)	9.7
(f)	4,6-DMDBT	Oxd./Sul. mass ratio (X_2)	3.0

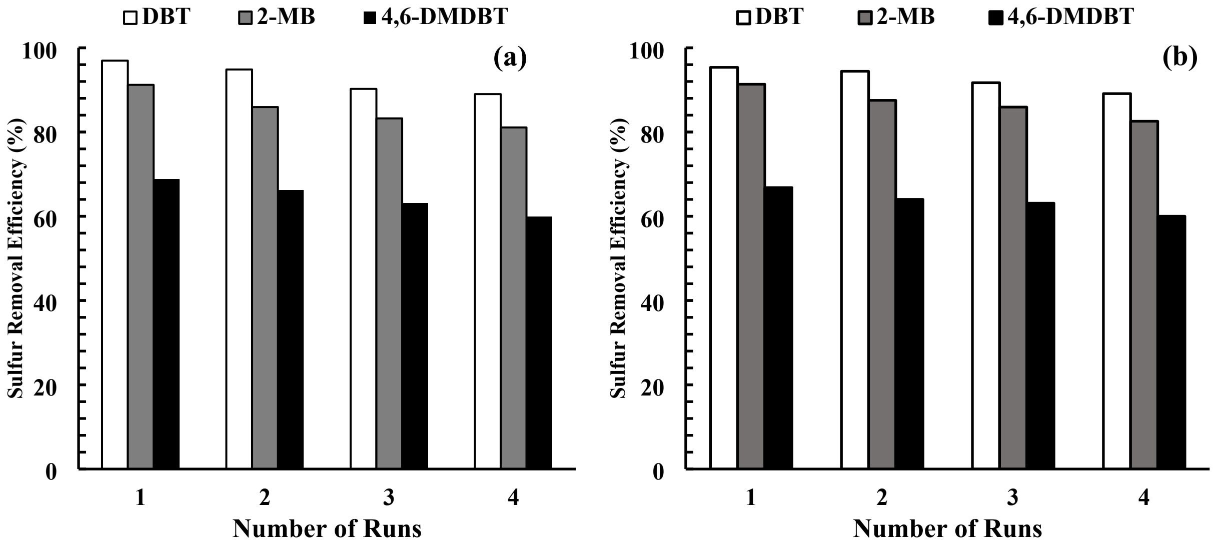

Figure S6. Sulfur removal yield on (a) UiO-66-OH and (b) UiO-66 MOF in four cycles at their optimum conditions
